# Optimizing the Synthesis of Titanium Carbide-Bismuth Oxide for Enhanced Antimicrobial Properties

**DOI:** 10.7759/cureus.67971

**Published:** 2024-08-27

**Authors:** Ojas Tamhane, Geetha A, Vasugi S, Balachandran S, Ilangovar I G K

**Affiliations:** 1 Department of Physiology, Saveetha Dental College and Hospitals, Saveetha Institute of Medical and Technical Sciences, Saveetha University, Chennai, IND

**Keywords:** antimicrobial activity, x-ray diffraction, stoichiometry, bismuth trioxide, mxene

## Abstract

Background

The two-dimensional MXene, known as titanium carbide (Ti₃C₂), is characterized by its substantial interlayer spacing, extensive surface area, hydrophilic nature, exceptional thermal stability, and outstanding electrical conductivity. These distinctive attributes render Ti₃C₂ an ideal candidate for detecting target analytes and immobilizing biomolecules. Bismuth oxide (Bi₂O₃), an essential compound of bismuth, frequently acts as a foundational element in bismuth chemistry. Its applications are diverse, from fireworks to oxygen gas sensors and solid oxide fuel cells, with particular emphasis on its behaviour under elevated temperatures and pressures. Notably, phase transitions to various polymorphs, which remain metastable at room temperature, have been documented under these conditions, indicating potential for numerous applications. Integrating MXene with Bi₂O₃ composites holds significant promise for advancements in energy-related electronics, sensing technologies, and photocatalytic processes.

Objective

To optimize the synthesis of titanium carbide-bismuth oxide (Ti₃C₂-Bi₂O₃) nanoparticles to enhance their antimicrobial activity by identifying the best synthesis conditions and assessing their effectiveness against various microbial pathogens.

Materials and methods

The preparation of Ti₃C₂ MXene involves dissolving lithium fluoride in hydrochloric acid, followed by Ti₃AlC₂ and stirring at 40°C for 48 hours. The resulting pellet is then dispersed in ultrapure water and centrifuged to obtain the MXene dispersion. Bi₂O₃ nanoparticles are prepared by preparing bismuth nitrate pentahydrate in nitric acid and adding sodium hydroxide to adjust the pH. The resulting white precipitate is filtered, washed, and dried before being calcined at 400°C for two hours to produce Bi₂O₃ nanoparticles. The Ti₃C₂-Bi₂O₃ composite is synthesized by adding Bi(NO₃)₃ solution to a 5 mg/mL Ti₃C₂Tx MXene solution. The reaction solution is heated to 160°C, and the resulting black powder is labelled as x% Bi₂O₃/MXene. The antimicrobial efficacy of the nanoparticles is assessed using the disk diffusion method. The zones of inhibition are measured and analyzed as indicators of antimicrobial activity.

Results

The scanning electron microscopy (SEM) analysis revealed the presence of Bi₂O₃ particles alongside Ti₃C₂​​​​​​​ nanosheets, while the X-ray diffraction (XRD) analysis and energy-dispersive X-ray spectroscopy (EDS) confirmed the high crystallinity of the compound. Furthermore, the compound was determined to be impurity-free and demonstrated antimicrobial properties.

Conclusion

The XRD analysis confirms the effective integration of various materials and the existence of crystalline phases. SEM provides insights into the morphology and organization of particles within sheets, whereas EDS assesses the elemental composition and its uniform distribution. These studies demonstrate the synthesis of Ti₃C₂-Bi₂O₃​​​​​​​ composites, suggesting their potential for usage in applications involving antimicrobial action.

## Introduction

The family of MAX phases and their derivatives, referred to as MXenes, is undergoing continuous growth in terms of both crystalline structures and compositional diversity. Recent developments have led to significant progress in the synthesis of novel MAX phases that incorporate ordered double-transition metals. This advancement has subsequently resulted in the emergence of new MXenes, which are distinguished by their increased chemical variety and structural complexity traits that are not typically found in other families of two-dimensional (2D) materials. Given the extensive range of elemental compositions, the capacity to adjust surface functionalities, the existence of various magnetic orders, and notable spin-orbit coupling, MXenes can be classified as genuinely multifunctional materials that facilitate highly correlated phenomena. Moreover, their large surface area, hydrophilic characteristics, adsorption potential, and high surface reactivity have attracted considerable attention for a variety of applications, such as catalysis, ion batteries, gas storage, and sensing technologies [1]. In light of the swift progress in MXene-related research and technology, it is crucial to update our comprehension of their varied properties and potential uses. As many theoretical predictions await experimental confirmation, this discussion will primarily concentrate on the observable physical and chemical properties of MXenes and examine how these attributes can be tailored for diverse applications. The distribution of surface functional groups on MXene is influenced by etching conditions, presenting an intriguing challenge [2].

The unique combination of enhanced hydrophilicity and metallic conductivity in MXene can be attributed to the presence of surface terminations (-O, -F, and -OH) along with transition metal carbide or nitride. MXene possesses outstanding physicochemical properties, including superior electrical conductivity, extensive surface areas, and notable optical and magnetic features, as well as impressive thermal and mechanical properties, indicating its considerable potential for future applications in the biological field [3]. The exceptional flexibility of MXene, coupled with its 2D morphology and layered structures, facilitates seamless interactions with other materials [4]. MXene exhibits both biocompatibility and biodegradability, facilitating its effective removal from the human body. These characteristics contribute significantly to the remarkable progress of MXene in nanomedicine applications, including cancer therapy, bioimaging, biosensing, and antibacterial solutions. Furthermore, the combination of MXene with graphene in a hybrid or composite form can fulfill numerous unmet requirements across various sectors, especially within medicine and biomedical engineering [5].

Nanomaterials made of bismuth oxide (Bi₂O₃​) have many useful properties, including a large band gap between 2 and 3.96 eV, a high refractive index, and strong photoluminescence. This oxide has numerous applications in the treatment of specific diseases as well as in the optimization of Bi₂O₃ nanostructures for use in electrical, medical, and biological sensors, among other pertinent fields. It offers scientific insights into the recombination sites of trapped charges associated with metastable defects within the lattice, contingent upon whether the entrapment process is influenced by thermal conditions [6]. The catalytic properties of Bi₂O₃ are significantly affected by the existence of oxygen vacancies. Consequently, even minor alterations in the lattice structure, caused by the presence of inclusion impurities, substituted ions, or surface defects at parts per million concentrations, demonstrate effective degradation of photocatalytic characteristics. Numerous investigations have been conducted on Bi₂O₃, focusing on optimizing reactions and modifying its structure (such as through metal doping or material hybridization) to enhance photoactivity and energy efficiency [7]. Bi₂O₃ ​​​​​is a multifunctional compound utilized in various applications. For the first time, a simple one-step polyol method has been employed to produce Bi₂O₃​​​​​ nanoparticles that are coated with biocompatible and hydrophilic D-glucuronic acid [8]. The potential of Bi₂O₃ as a contrast agent for computed tomography (CT) was evaluated through an analysis of its X-ray attenuation properties. Bi₂O₃ is a multifaceted material that has been investigated for its possible applications in bone tissue engineering. The nanostructures of Bi₂O₃ were produced via the electrochemical anodization of a bismuth film in an oxalic acid electrolyte [9].

Nanoparticles can serve as effective carriers for targeted drug delivery to specific locations within the body. Additionally, Bi₂O₃​​​​​ has demonstrated photothermal properties, allowing it to convert light into thermal energy. This characteristic has been investigated for use in photothermal therapy, where Bi₂O₃ nanoparticles are precisely directed to tumour sites and subsequently subjected to infrared light, resulting in the effective destruction of cancer cells [10]. While bismuth is generally considered safe, as previously mentioned, extended exposure to it may lead to negative consequences for human health. Bi₂O₃​​​​​ presents a valuable option due to its ability to absorb visible light, excellent chemical stability, non-toxic nature, and affordability [11]. We will synthesize Bi₂O₃ ​​​​​​modified with Bi₂O₃, which will be characterized using various analytical techniques. The electrochemical energy storage capabilities of titanium carbide (Ti₃C₂) ​​are attributed to its 2D structure, outstanding electrical properties, and biocompatibility. Composites of Ti₃C₂-Bi₂O₃ ​​​​​​​hold promise for applications in energy-related electronics, sensing technologies, and photocatalytic processes [12]. The study investigates the antimicrobial properties of Ti₃C₂-Bi₂O₃ nanoparticles, which show promising effectiveness against various bacterial and fungal strains, and explores methods to enhance synthesis, processing, and characterization techniques for medical applications.

## Materials and methods

Materials

Titanium aluminium carbide (Ti₃AlC₂) was purchased from Sigma Aldrich, St. Louis, USA. Hydrofluoric Acid (HF) and bismuth nitrate pentahydrate [Bi(NO₃)₃·5H₂O] are from SRL Chemicals, Mumbai, India.

Preparation of Ti₃C₂ MXene

A total of 2.0 g of lithium fluoride was gradually dissolved in 40 mL of 9 M hydrochloric acid (HCl). The solution was then stirred for 30 minutes to ensure the complete dissolution of the solids. Next, 1 g of Ti₃AlC₂ was slowly introduced into the solution, and the mixture was stirred at 40°C for 48 hours. After the reaction was complete, the remaining lithium fluoride was removed by washing with 1 M HCl. The solids were then washed and centrifuged multiple times with deionized water until the solution's pH exceeded 6. The resulting pellet was dispersed in 100 mL of ultrapure water and subjected to sonication in an argon atmosphere for two hours. Following sonication, the dispersion was centrifuged for 30 minutes at 3500 rpm to obtain the supernatant liquor, which contained the MXene dispersion.

Preparation of Bi₂O₃ nanoparticles

Bi(NO₃)₃·5H₂O serves as the precursor for Bi₂O₃ nanoparticles. A solution of Bi(NO₃)₃·5H₂O is prepared in nitric acid (HNO₃) and stirred until fully dissolved. Sodium hydroxide (NaOH) is then added dropwise to this solution while maintaining constant stirring, adjusting the pH to approximately 9, which results in the formation of a white precipitate of bismuth hydroxide [Bi(OH)₃]. The precipitate is subsequently filtered, washed with deionized water, and dried at 100°C. Finally, the dried Bi(OH)₃ is calcined at 400°C for two hours in the air to produce Bi₂O₃ nanoparticles.

Synthesis of Ti₃C₂-Bi₂O₃ composite

A total of 0.24 g of Bi(NO_3_)_3_·5H_2_O was dissolved in a mixture of 17 mL glycol and 34 mL ethanol to prepare a 0.01 mol/L Bi(NO_3_)_3_ solution. Around 2 mL of this Bi(NO_3_)_3_ solution was added dropwise to 3.05 mL of a 5 mg/mL Ti₃C₂Tx MXene solution. After the addition, the mixture was subjected to ultrasound for 30 minutes and then transferred to a Teflon-lined stainless steel autoclave, which was heated to 160°C. After two hours, the reaction solution was allowed to cool to room temperature and was then washed with ultrapure water. The resulting black powder was collected by centrifugation and freeze-drying. This material was labelled as x% Bi₂O₃/MXene, where x indicates the molar ratio of Bi to C (i.e., 5.5%, 11%, 22%, and 44%). The corresponding concentrations of the Bi(NO_3_)_3_ solution were 5 mmol/L, 10 mmol/L, 20 mmol/L, and 40 mmol/L, respectively. For catalyst application, 5 mg of the catalyst was mixed with 1 mL of Nafion-ethanol solution and 200 μL of the ink suspension was uniformly applied to carbon paper (1×1 cm²), which was then dried in a vacuum for two hours before use.

Antimicrobial activity

The disk diffusion method was employed to assess the antimicrobial efficacy of Ti₃C₂-Bi₂O₃ nanoparticles. Initially, 50 mg of the nanoparticles were dissolved in 2.5 mL of ethanol and subsequently sterilized using a Millipore filter with a pore size of 0.22 mm from Merck (Darmstadt, Germany). This solution was then applied to sterile filter paper discs, each measuring 8 mm in diameter, to achieve the desired concentration. A base layer of 10 mL of Mueller-Hilton agar medium was poured into sterilized Petri dishes. The filter paper discs, containing Ti₃C₂-Bi₂O₃ nanoparticles at a concentration of 10 mg/mL, were placed on the agar surface. For comparison, filter paper discs with 20 µg of ketoconazole (for fungi) and 20 µg of tetracycline (for bacteria) were included as a positive control, with the blank dimethyl sulfoxide (DMSO) serving as the negative control. The plates were kept at 5°C for two hours to allow for the diffusion of the nanoparticles, followed by incubation at 35°C for 24 hours for bacterial strains, specifically *Enterococcus faecalis* and *Streptococcus mutans*, and at 25°C for fungal strains, including *Candida albicans* and *Candida parapsilosis.* The zones of inhibition were measured using a Vernier calliper, recorded, and analyzed as indicators of antimicrobial activity.

## Results

X-ray diffraction (XRD) analysis

**Figure 1:**
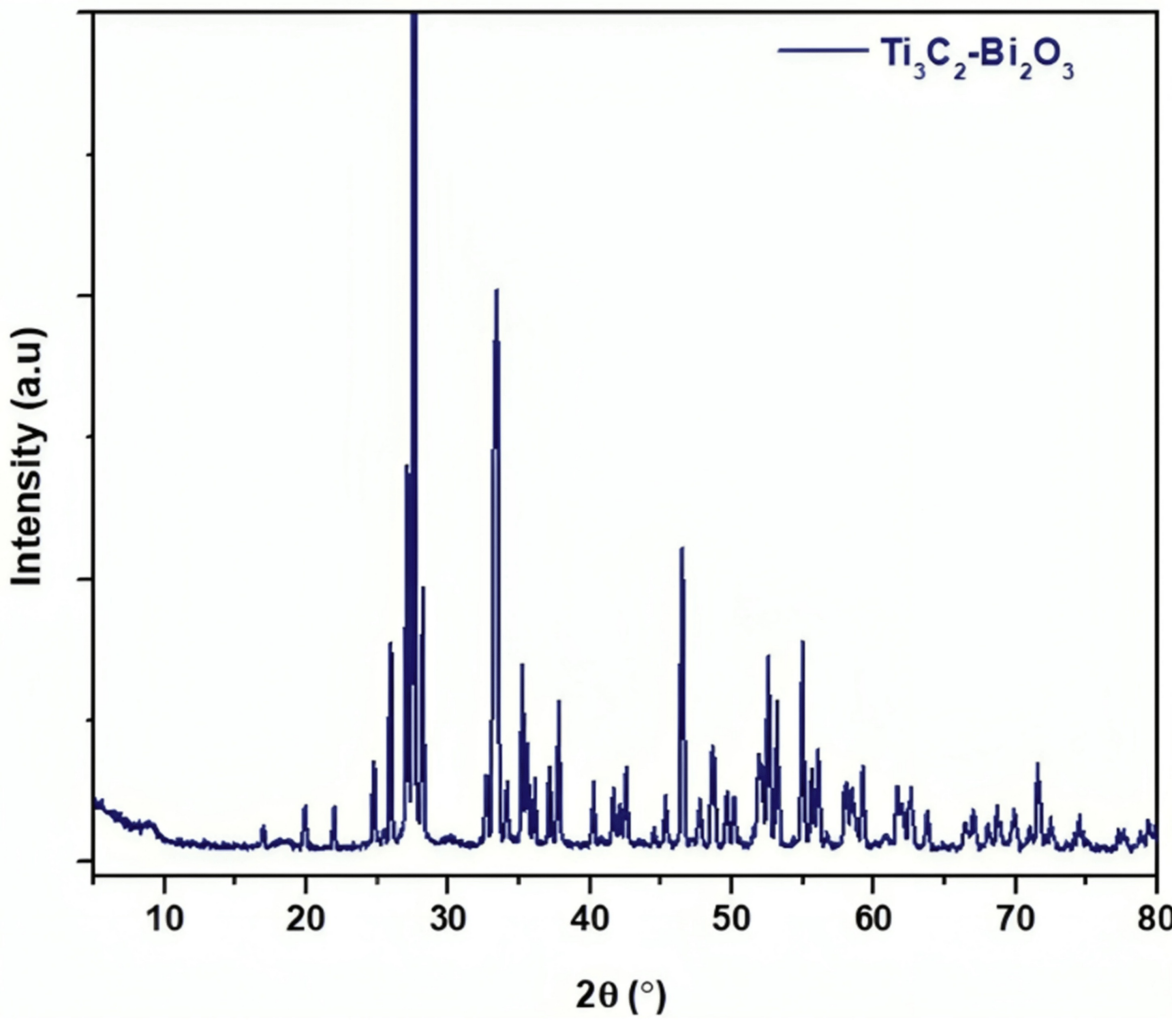
XRD Analysis of the Ti₃C₂-Bi₂O₃ XRD: X-ray diffraction; Ti₃C₂-Bi₂O₃: titanium carbide-bismuth oxide

The XRD pattern obtained for the Ti₃C₂-Bi₂O₃ sample offers significant insights into its crystalline architecture. The pattern exhibits peaks at various 2θ angles, which correspond to the atomic spacing within the crystalline structure. Specific peaks at designated angles signify different crystallographic planes present in the material. For example, a notable peak near 27° (2θ) is likely attributed to the (002) planes of Ti₃C₂, a hallmark of MXenes. Additional peaks observed between 20° and 30° indicate the presence of layered structures, while peaks ranging from 30° to 70° may be linked to both Ti₃C₂ (as per the Joint Committee on Powder Diffraction Standards (JCPDS) card 41-1049) and Bi₂O₃ phases (referencing JCPDS file #41-1449). The intensity of these peaks reflects the degree of constructive interference, which is indicative of the atomic density and orientation of the crystal planes. More pronounced peaks suggest higher atomic density or superior alignment with the X-ray beam. The pattern signifies a composite material comprising both Ti₃C₂ and Bi₂O₃ phases. Typically, Ti₃C₂ displays peaks around 9° for the (002) plane, and any shifts in peak positions may indicate alterations in interlayer spacing due to intercalation or other modifications. Peaks associated with Bi₂O₃ should align with standard patterns for Bi₂O₃, thereby confirming its presence. Sharp peaks in the XRD pattern denote high crystallinity, suggesting that the material possesses a well-ordered crystal structure. Conversely, broader peaks would imply the existence of more amorphous or poorly crystalline regions. The XRD pattern illustrates that the Ti₃C₂-Bi₂O₃ composite maintains distinct phases with commendable crystallinity, thereby validating the successful synthesis of the material and offering valuable insights into its structural characteristics (Figure 1).

Energy-dispersive X-ray spectroscopy (EDS) analysis

EDS analysis is a technique that is used to determine and quantify the elements in the Ti₃C₂-Bi₂O₃ sample. This method gives information about the specific elemental composition of the sample by identifying the characteristic X-ray emissions originating from each element. An EDS analysis of Ti₃C₂-Bi₂O₃ reveals that the elements titanium (Ti) and carbon (C) from Ti₃C₂, along with bismuth (Bi) and oxygen (O) from Bi₂O₃, are the main components. The EDS spectrum shows the X-ray peaks for each of these elements, allowing for accurate quantification.

**Figure 2:**
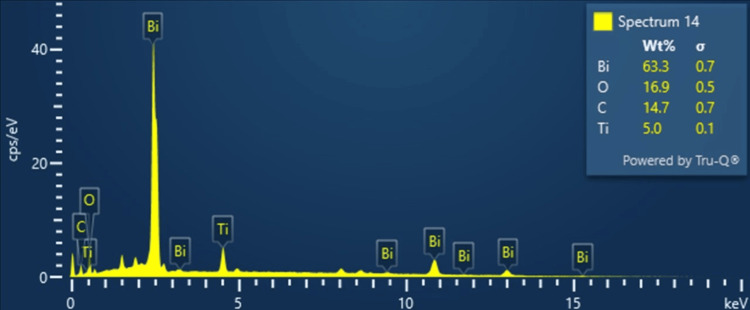
EDS Analysis of the Ti₃C₂-Bi₂O₃ EDS: energy dispersive X-ray spectroscopy; Ti₃C₂-Bi₂O₃: titanium carbide-bismuth oxide

As the spectrum shows, the sample has the following composition: 5.0% Ti, 14.7% C, 16.9% O, and 63.3% Bi. The total weight percentage of Ti₃C₂-Bi₂O₃ in the sample is registered as 99.99%, which means the synthesized compound is of a high level of purity with the presence of impurities being almost negligible (Figure 2). 

Scanning electron microscopy (SEM) analysis

**Figure 3:**
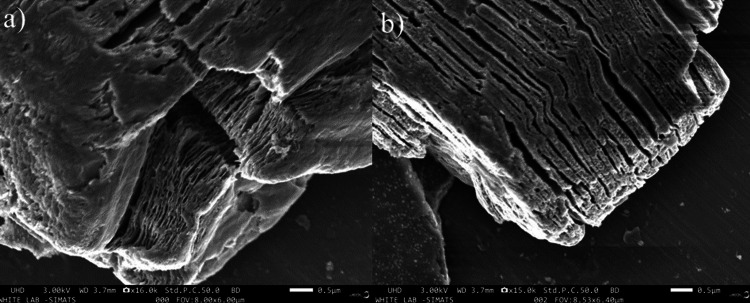
SEM Analysis of Ti₃C₂-Bi₂O₃ at (a) 16.0k Resolution and (b) 15.0k Resolution SEM: scanning electron microscopy; Ti₃C₂-Bi₂O₃: titanium carbide-bismuth oxide

The SEM images of Ti₃C₂-Bi₂O₃ were analyzed using an ultrahigh-definition (UHD) microscope operating at a voltage of 3.00 kilovolts (kV) and a working distance (WD) of 3.7 mm, with a resolution of 0.5 µm. The morphology of the sample was examined using SEM techniques. As illustrated in Figure 3a, at a resolution of 16.0k, the HF-etched Ti₃C₂ MXene exhibits a well-stacked, sheet-like structure that is effectively delaminated. The SEM images of Ti₃C₂ MXene reveal a well-organized, layered arrangement of the nanosheets. These nanosheets are notably thin, consist of multiple layers, and have distinct, sharp edges, as depicted in Figure 3b at a resolution of 15.0k. This observation confirms the high quality of the synthesized Ti₃C₂ MXene. The images clearly show that the small spherical particles on the surfaces of Ti₃C₂ MXene are Bi₂O₃. The high specific surface area of Ti₃C₂ MXene facilitates the adsorption of Bi₂O₃ nanoparticles, resulting in a uniform distribution of Bi₂O₃ across the Ti₃C₂ MXene. The SEM images reveal that the synthesized Ti₃C₂-Bi₂O₃ has a crystalline morphology.

Antimicrobial activity

The antimicrobial activity of the compound Ti₃C₂-Bi₂O₃ against various bacterial and fungal strains quantified by the zone of inhibition in mm. For *C. albicans*, no inhibition was observed at either 50% or 100% concentration of the compound, although the positive control exhibited a zone of inhibition measuring 31 ± 2.52 mm. *C. parapsilosis *showed no inhibition at 50% concentration, but at 100% concentration, an inhibition zone of 12 ± 1.53 mm was observed; the positive control for this strain demonstrated a zone of inhibition of 24 ± 1.53 mm. *E. faecalis* exhibited no inhibition at either 50% or 100% concentration, with the positive control showing a zone of inhibition of 29 ± 1.53 mm. For *S. mutans*, inhibition zones of 10 ± 0.58 mm at 50% concentration and 18 ± 1.75 mm at 100% concentration were observed, while the positive control indicated a zone of inhibition of 26 ± 1.53 mm. The compound showed no inhibitory effect on *C. albicans* and *E. faecalis* at either concentration tested. However, *C. parapsilosis* exhibited some inhibition at 100% concentration. *S. mutans* demonstrated inhibition at both concentrations, with a larger zone of inhibition at the higher concentration of 100% (Table 1).

**Table 1:** Zone of Inhibition by Ti₃C₂-Bi₂O₃ on Different Bacterial and Fungal Strains The positive control confirms that the assay can detect antimicrobial activity, while the negative control confirms that the observed inhibition in other samples is due to the test compounds and not due to any artefact or contamination. Positive control for *C. albicans* and *C. parapsilosis* is ketoconazole and *E. faecalis* and *S. mutans *is tetracycline. Negative control for *C. albicans, C. parapsilosis, E. faecalis* and *S. mutans* is DMSO (dimethyl sulfoxide).

S. No.	Bacterial and Fungal Strains	Titanium Carbide-Bismuth Oxide (Ti_3_C_2_-Bi_2_O_3_)		Positive Control (mm)	Negative Control (mm)
		Zone of Inhibition (mm)			
		Concentration 50%	Concentration 100%		
1	Candida albicans	-	-	31 ± 2.52	-
2	Candida parapsilosis	-	12 ± 1.53	24 ± 1.53	-
3	*Enterococcus faecalis*	-	-	29 ± 1.53	-
4	Streptococcus mutans	10 ± 0.58	18 ± 1.75	26 ± 1.53	-

## Discussion

MXenes have emerged as a promising material characterized by a substantial surface area, excellent thermal and electrical conductivity, and an adjustable band gap, exhibiting remarkable hydrophilicity and stability. The adsorption and reduction capabilities of MXene-based 2D nanomaterials render them effective photocatalysts for the degradation of organic pollutants [13]. Mashtalir et al. demonstrated that XRD calibration can be performed using mixtures with established ratios of pure MXene and the original MAX phase as reference samples, thereby illustrating the influence of time and temperature on the yield of MXene. Furthermore, the removal of "A" layers from the MAX phase leads to the formation of multilayer structures, attributed to the strong hydrogen bonding that promotes stacking between individual sheets. To achieve complete delamination into single-layer MXene, additional procedures are necessary to enhance the layer spacing at the nanometer scale. Consequently, XRD serves as an effective method for monitoring variations in layer spacing, which is essential for optimizing the yield of single-layer MXenes [14]. Naguib et al. demonstrated that the treatment of Ti_3_CNTx with tetrabutylammonium hydroxide (TBAOH) for a duration of two hours resulted in a notable shift in the (002) peak from a 2θ value of 8.26° to 4.57°. This alteration indicates an increase in the c-LP from 21.4 Å to 38.6 Å. Following an extended mixing period of four hours, the (002) peak further shifted to a 2θ of 4.5°, which corresponds to a c-LP of 39.2 Å. These findings confirmed that small molecules penetrate between the MXene layers, thereby establishing a fundamental method for weakening the interlayer bonds, which is essential for achieving large-scale delamination [15]. Rivenet et al. examine the various allotropic forms of Bi_2_O_3_ and how these forms are influenced by the temperature range employed during the synthesis process. Following decomposition at 500°C, the resulting oxide exhibits peaks that have been systematically assigned and indexed according to JCPDS file #41-1449. The oxide identified corresponds to the α-Bi_2_O_3_ allotrope, characterized by its crystallographic parameters, which include linear dimensions of a = 5.8499(3), b = 8.1698(4), and c = 7.5123(3). Additionally, the angular parameters are defined as α = 90°, β = 112.988(4)°, and γ = 90° [16]. EDS, also known as EDAX, is an X-ray technique employed for the detection and analysis of elemental composition within the Ti₃C₂-BiOCl sample. The primary constituents of Ti₃C₂ are titanium and carbon. The EDS spectrum reveals distinct X-ray peaks that are indicative of the elements present in the sample, and the intensities of these peaks can be used to ascertain the relative quantities of each element. EDS mapping results indicate a consistent distribution of four elements: C, O, Ti, Bi, and chloride (Cl) within the Ti₃C₂​​​​​​​-BiOCl compound. The weight percentages of these elements are recorded as 32.45% for C, 27.12% for O, 25.64% for Ti, 14.79% for Bi, and 0.0% for Cl. The total weight of the Ti₃C₂​​​​​​​-BiOCl compound sums to 100%, which underscores its purity and the absence of impurities in the synthesized material. The EDS analysis further corroborates that no extraneous materials are present, confirming that the Ti₃C₂​​​​​​​-BiOCl is free from impurities [17]. The typical morphology of exfoliated MXene is characterized by a flat and smooth layered structure with sharp edges, which indicates the complete etching of MAX (Ti_3_AlC_2_) using HF as the etchant.

In contrast, CuCr_2_O_4_ particles, which vary in size, shape, and compositional ratio, are uniformly agglomerated onto the Ti₃C₂​​​​​​​Tx nanoflakes, resulting in a sandwich-like morphology. The formation of the nanocomposite induces structural distortion, which enhances the surface-to-volume ratio and facilitates additional diffusion pathways for electrolyte ions. Consequently, this leads to a uniform distribution of CuCr_2_O_4_ nanoparticles across the interlayers and surface of Ti₃C₂​​​​​​​Tx, thereby improving overall conductivity. The effective intercalation of CuCr_2_O_4_ nanoparticles within the nanocomposite structure yields an average grain size of 1.53 nm, as determined using ImageJ software through the formula for grain size calculation, which is the length of the line divided by the number of grains counted. The presence of Ti₃C₂​​​​​​​Tx confirms a distinct multilayer structure, indicating that the aluminium layer has been selectively removed from the Ti_3_AlC_2_ phase [18]. Arabi Shamsabadi et al. conducted a study to evaluate the antibacterial efficacy of MXene nanosheets of varying sizes against *Escherichia coli *and *Bacillus subtilis *under dark conditions, thereby eliminating the potential influence of the photothermal effects associated with the MXene nanoparticles. Their findings revealed that the nanosheets induced bacterial DNA leakage and caused the dispersion of bacterial cells within a time frame of three hours [19]. Beyond the observational analysis of this antimicrobial phenomenon, Jastrzebska et al. conducted a comprehensive investigation into the structures of Ti_2_C and Ti₃C₂​​​​​​​ MXene, exploring the correlation between their atomic configurations and antimicrobial characteristics. Their findings indicated that there were no discernible differences in surface chemistry between Ti_2_C and Ti₃C₂​​​​​​​ MXene. Notably, while Ti_2_C MXene did not influence bacterial survival, Ti₃C₂​​​​​​​ MXene demonstrated significant antibacterial properties. This observation was attributed to the identical chemical composition at the atomic level, despite variations in stoichiometry. Furthermore, in addition to their inhibitory effects on commonly encountered bacteria, MXenes were also found to exhibit antifungal properties [20]. In this context, Lim et al. investigated the antifungal characteristics of Ti₃C₂​​​​​​​Tx MXene, employing inverted contrast microscopy to demonstrate that a significant quantity of mycelium and spores was present in the control group that lacked MXene [21]. In contrast, the experimental group that received treatment with delaminated Ti₃C₂​​​​​​​Tx (d-Ti₃C₂​​​​​​​Tx) MXene exhibited a notable inhibition of fungal growth. This observation prompted the authors to deduce that the nanosheets of d-Ti₃C₂​​​​​​​Tx MXene effectively achieved this result by interfering with the structural integrity of the fungal hemispheres [22].

Limitations

The optimization of Ti₃C₂-Bi₂O₃ nanoparticles for enhanced antimicrobial properties is challenging due to their complex synthesis process, which requires precise control of conditions like temperature and pH. Scaling up from laboratory to industrial production may also pose difficulties, potentially limiting practical applications. The stability of the nanoparticles is another concern, as they may aggregate or degrade over time, affecting their effectiveness. Limited solubility in aqueous solutions can impact the uniform distribution and efficacy of the nanoparticles. Toxicity to human cells and the environment needs thorough investigation. Antimicrobial activity may vary based on microbial strains, and accurate characterization requires sophisticated equipment. New antimicrobial agents must undergo rigorous testing and regulatory approval before medical use. Proper storage and handling are essential for maintaining effectiveness, but they can be challenging. Limited existing research makes it difficult to predict the nanoparticles' behaviour in various applications. Overuse of antimicrobial agents, including nanoparticles, may lead to the development of resistant microbial strains, reducing their efficacy over time.

## Conclusions

The synthesis of Ti₃C₂-Bi₂O₃ nanoparticles holds significant promise for enhancing antimicrobial properties. Through precise control of synthesis conditions and thorough characterization, Ti₃C₂-Bi₂O₃ nanoparticles have demonstrated effective antimicrobial activity against a range of bacterial and fungal pathogens. However, challenges such as scalability, stability, solubility, and potential toxicity need to be addressed to ensure practical applications. Continued research is essential to refine synthesis methods, improve nanoparticle stability, and evaluate long-term safety. Ultimately, advancements in these areas could lead to the development of highly effective antimicrobial agents with broad applications in medical and environmental fields.
